# Biosynthesis and Characterization of Biogenic Tellurium Nanoparticles by Using *Penicillium chrysogenum* PTCC 5031: A Novel Approach in Gold Biotechnology

**Published:** 2018

**Authors:** Hamed Barabadi, Farzad Kobarfard, Hossein Vahidi

**Affiliations:** a *Department of Pharmaceutical Biotechnology, School of Pharmacy, Shahid Beheshti University of Medical Sciences, Tehran, Iran. *; b *Student Research Committee, School of Pharmacy, Shahid Beheshti University of Medical Sciences, Tehran, Iran. *; c *Department of Medicinal Chemistry, School of Pharmacy, Shahid Beheshti University of Medical Sciences, Tehran, Iran. *

**Keywords:** Tellurium nanoparticles, Biosynthesis, Characterization, Penicillium chrysogenum, Gold biotechnology

## Abstract

Production of nanoparticles has been attractive by biological based fabrication as an alternative to physical and chemical approaches due to exceeding need to develop safe, reliable, clean and eco-friendly methods for the preparation of nanoparticle for pharmaceutical and biomedical applications. In the present study, biogenic tellurium nanoparticles (TeNPs) were successfully prepared using potassium tellurite (K_2_TeO_3_, 3H_2_O) via an eco-friendly and simple green approach by exploiting extracellular enzymes and biomolecules secreted from *Penicillium chrysogenum* PTCC 5031 at room temperature for the first time. The biofabricated TeNPs were characterized by Atomic Force Microscope (AFM), Scanning Electron Microscopy (SEM), Dynamic Light Scattering (DLS), Energy Dispersive X-ray spectroscopy (EDX), and Fourier Transform Infrared (FT-IR) spectrum. The AFM and SEM images revealed that the TeNPs were fairly uniform in size with a spherical shape and superior monodispersity. Furthermore, the DLS indicated that the average hydrodynamic diameter of TeNPs was around 50.16 nm and polydispersity index (PdI) of 0.012. The EDX results depicted that TeNPs display an absorption peak at 3.8 keV, indicating the presence of the elemental tellurium. Additionally, the FT-IR analysis of TeNPs exhibited the presence of possible functional groups that may have a role as bioreducers and capping agents. Overall, the results strongly suggested that *P. chrysogenum* can be a potential nanofactory for the preparation of TeNPs due to several advantages including non-pathogenic organism, fast growth rate, and high capacity of elemental ions reduction, as well as facile and economical biomass handling.

## Introduction

Biotechnology possesses a wide range of biological applications in different fields of science. Hence, one of the beneficial classifications of biotechnology scopes refers to DaSilva who proposed a rainbow in the year 2004 including ten colors (red, yellow, blue, green, brown, dark, purple, white, gold, and gray) based on different biotechnology fields to simplify its complexity. According to this codification, the studies in the scope of nanobiotechnology are sub-classified as a part of a larger group named gold biotechnology (1-3). Nanobiotechnology refers to the intersection of biotechnology and nanotechnology and one of the interesting areas in nanobiotechnology is biofabrication of environmentally friendly metallic nanoparticles smaller than 100 nm (4-6). These nanoparticles unlike bulk materials display specific physical, chemical, and biological properties owing to their large surface to volume ratio along with large surface energy (7, 8). Up to now, a number of microorganisms containing bacteria, fungi, yeast, plant extracts, and even algae have been reported for biofabrication of nanoparticles with different shapes and sizes as possible eco-friendly alternatives to conventional chemical and physical methods (5, 9-19). Significantly, the size, shape, chemistry, and controlled dispersities of nanoparticles have an influence on their unique natures (20). Remarkably, eco-friendliness, energy efficiency, cost effectiveness, simplicity, and compatibility for biomedical applications are the superiorities of biological approach for production of nanoparticles over conventional synthesis owing to their high cost resulting from the need to high pressure, energy, and temperature as well as their demand for toxic reducers and/or stabilizers that avoid their use for pharmaceutical applications (21-23). Tellurium is a metalloid rare element in the nature. The word "tellurium" is originated from the Latin word "tellus" which means "earth" (24). Particularly, tellurium as a crystalline, lustrous, brittle, and silver-white element belongs to the group named chalcogens (24, 25). Additionally, its discovery is attributed to a Transylvanian mining chemist in 1782, who was studying gold-containing ores (26, 27). Besides, tellurium with the abundance ranging 0.0001-0.02 ppm, is assumed as the 72nd most abundant element founds often combined with other metals in the earth's crust in four oxidation states including -2 (H_2_Te), +2 (TeO_2_^2-^), +4(TeO_3_^2-^), and +6 (TeO_4_^2-^) of which tellurite (TeO_3_^2−^) is the most abundant form. Interestingly, it was reported that more than 500 mg of tellurium is traced in human body mainly (24). Moreover, the antibacterial activity of tellurium has been demonstrated. Impressively, in recent years an enormous growth of interest in biomedical and pharmaceutical applications of TeNPs has been seen. For example, Mirjani *et al* 2015 reported a significant reduction in cholesterol and triglyceride levels in mice blood samples by oral intake of TeNPs enriched via probiotics (28). Additionally, Huang *et al* 2016 reported antioxidant activity of TeNPs as well as significant in vitro anticancer activity against breast adenocarcinoma (MCF-7) cells with IC_50_ value of 5.52 ± 0.89 μg/mL, while it exhibited no toxicity against renal tubular epithelial (HK-2) cells at the same concentration. Considerably, it was reported that TeNPs exhibited less than 5% hemolysis in erythrocytes up to 15 μg/mL representing excellent hemocompatibility (29). Significantly, the extent to which microorganisms can play a role in biofabrication of nanoparticles continues to be an issue of interest to researchers. While, there is a growing empirical evidence that different bacteria can successfully produce TeNPs in various morphologies (24). It is still unclear whether fungi can biofabricate TeNPs or not due to the advantages of fungi compared to bacteria including effectively extracellular enzymes secretion, biomass employing facility like simply separation of biomass by using filtration because of their filamentous structures (30). Consequently, the objective of the present study was preparation of TeNPs in a biological approach by using *P. chrysogenum* PTCC 5031 for the first time. 

## Experimental

*Chemicals*

Potassium tellurite (K_2_TeO_3_, 3H_2_O), yeast extract, sucrose, and other chemical reagents were purchased from Merck, Germany. Moreover, the lyophilized vial of *P. chrysogenum* PTCC 5031 was purchased from Iranian Research Organization for Science and Technology, Tehran, Iran.

*Extracellular biosynthesis of tellurium nanoparticles*

Initially, the fungus *P. chrysogenum* PTCC 5031 was cultured on modified fluid Czapek-Dox broth incorporating 21g sucrose and 3g yeast extract in 1000 mL distilled water and incubated on a rotary shaker (JAL TAJHIZ®, JTSL 40, Iran) at 120 rpm for seven days at 28°C. Further, the culture was centrifuged at 10,000 rpm by centrifuge (HETTICH®, ROTINA 380R, Germany) for 10 minutes to separate the mycelia from the supernatant. Subsequently, a stock of 1000 mL of a solution containing 1 mM of K_2_TeO_3_, 3H_2_O was prepared using double distilled water and was maintained at aseptic conditions after autoclaving. Following this, 100 mL of prepared potassium tellurite solution was added to 100 mL of supernatant with final pH value of 9 and incubated again at 120 rpm for five days at 28 °C (total concentration of K_2_TeO_3_, 3H_2_O = 0.5 mM). Concomitantly, as a negative control, 100 mL of potassium tellurite solution containing 1 mM of K_2_TeO_3_, 3H_2_O was added to 100 mL of fluid Czapek-Dox broth which was not cultured with fungus and incubated at the same condition for comparison. Thereafter, the biofabricated TeNPs were centrifuged at 15,000 rpm by centrifuge (HETTICH®, MIKRO 200, Germany) for 15 min to separate TeNPs from the reaction medium. Subsequently, to enhance TeNPs purification, the settled TeNPs were washed thrice with deionized double distilled water. Finally, TeNPs dried at 40 °C and were stored carefully in vials for further analysis.

*Characterization of tellurium nanoparticles*

The macroscopic determination of TeNPs formation was observed not only by the color change of the solution from pale yellow to dark, but also by Tyndall effect which is a phenomenon in which light is scattered by colloidal particles causing a laser beam to become visible by illuminating in its path (31) suggesting the convert of tellurium ions to their nano-forms. Moreover, to determine the size and distribution of TeNPs, Dynamic Light Scattering (DLS) and Polydispersity Index (PdI) were carried out as well as zeta potential to determine the electrostatic stability of TeNPs by a Zetasizer Nanoparticle Analyzer using Zetasizer 3600 at 25 °C with a scattering angle of 90° (Malvern instruments, UK). Additionally, the analysis of the surface and shape characteristics of TeNPs were determined by Scanning Electron Microscope (SEM) (MIRA3 model, Czech Republic) operated at 15 kV coupled with Energy Dispersive X-ray (EDX) analysis as well as Atomic Force Microscope (AFM) (JPK, NanoWizard II model, Germany) under ambient conditions in non-contact mode by employing silicon nitride tips with varying resonance frequencies at a linear scanning rate of 0.5 Hz. To analyze the size distribution of synthesized TeNPs through SEM and AFM, the diameter of fifty particles was measured randomly by using ImageJ software. Remarkably, EDX analytical technique was conducted in the spot-profile mode by focusing the electron beam onto an area on a surface coated with TeNPs for the elemental characterization. Furthermore, to identify the possible bio-molecules capping the surface of TeNPs, Fourier Transform Infrared (FT-IR) spectrum was conducted by mixing the TeNPs with potassium bromide at 1:100 ratios and compressed to a 2-mm semi-transparent disk for 2 min. The FT-IR spectrum was carried out over the wavelength range of 400-4000 cm^−1^ (Agilent, Cary 630 model, US).

## Results

**Figure 1:**
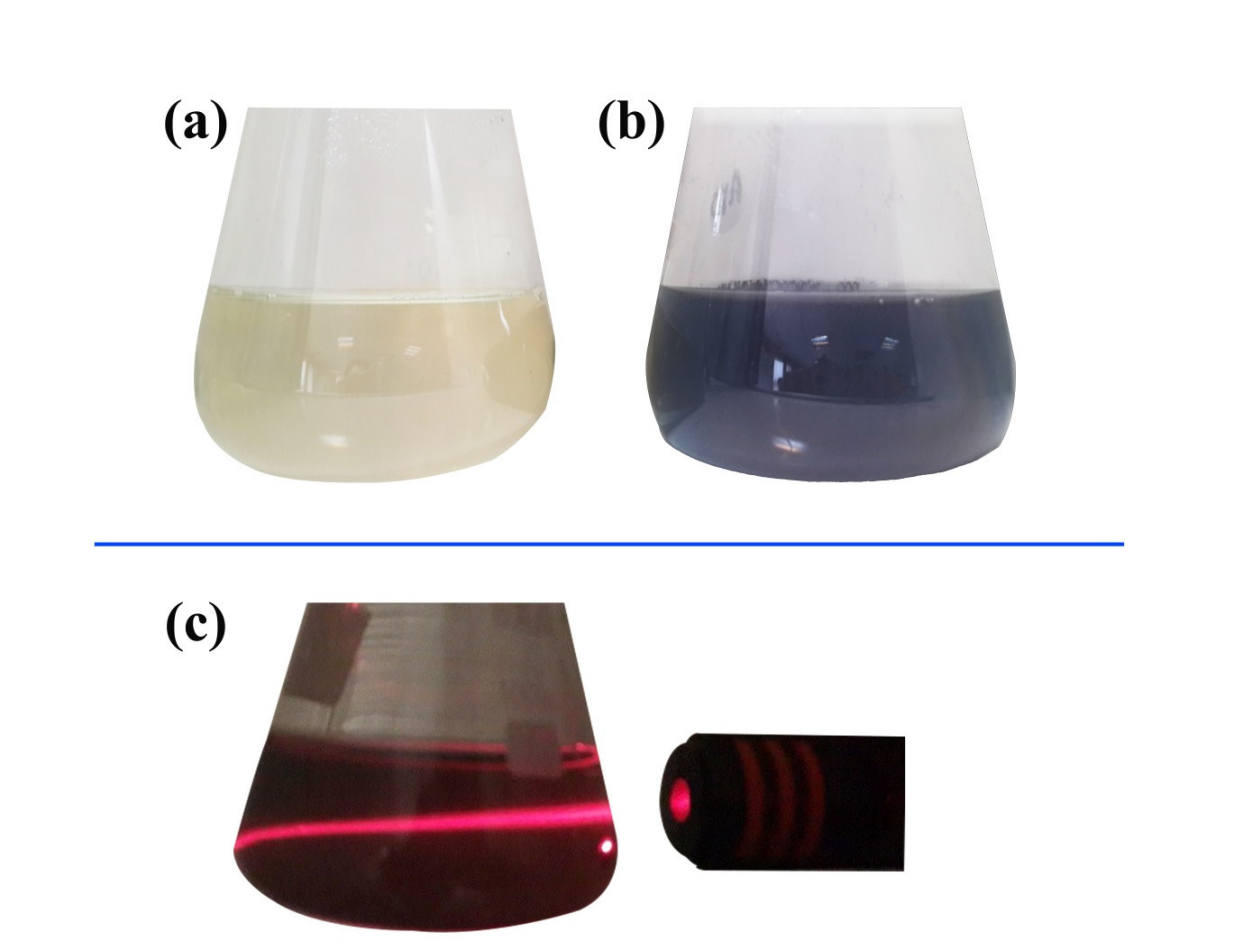
The optical photograph of color change from pale yellow (a) to gray black (b), and visible laser beam path due to the Tyndall effect (c).

**Figure. 2:**
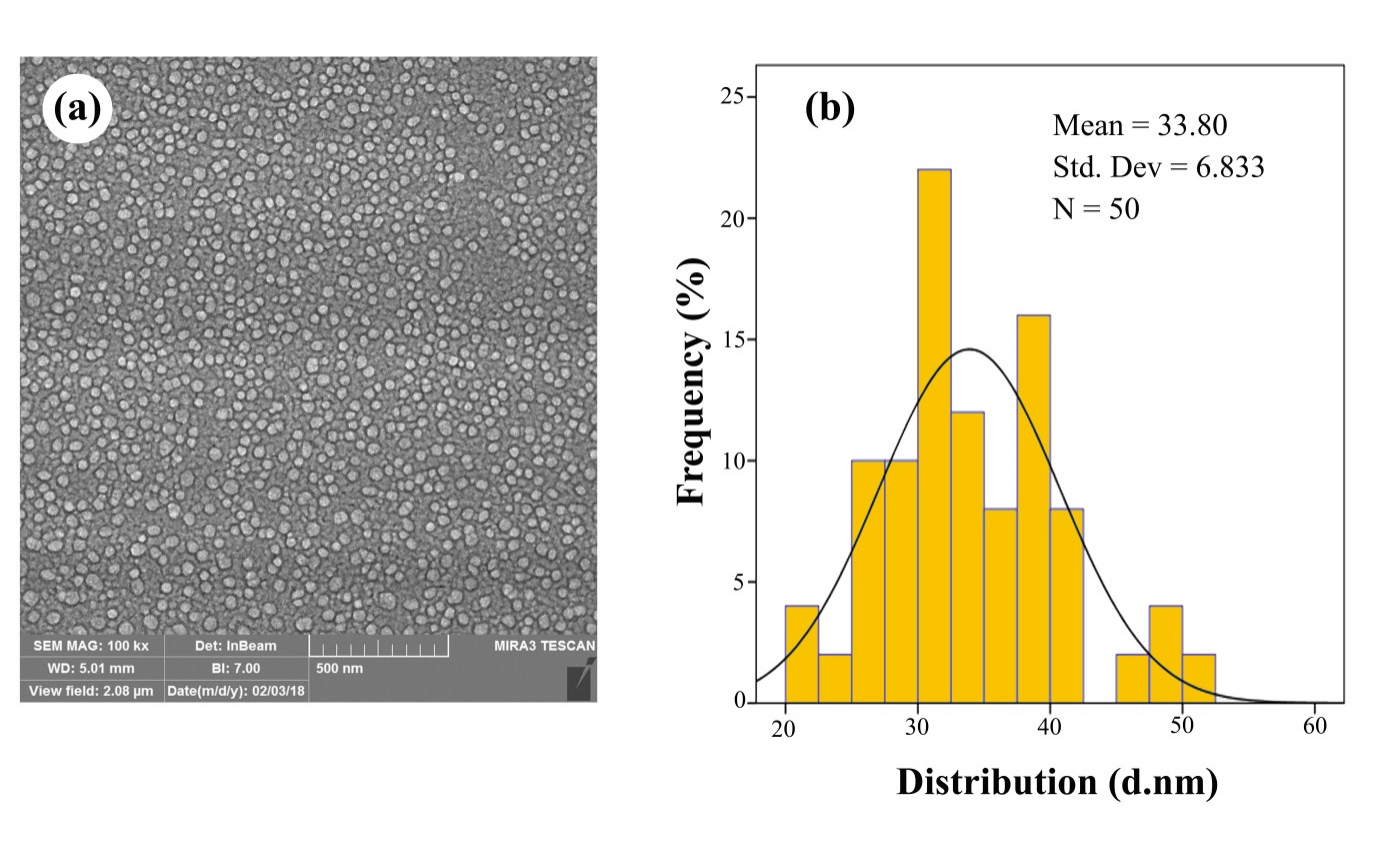
SEM image (a) and size distribution (b) of TeNPs produced by *P. chrysogenum *PTCC 5031

**Figure 3:**
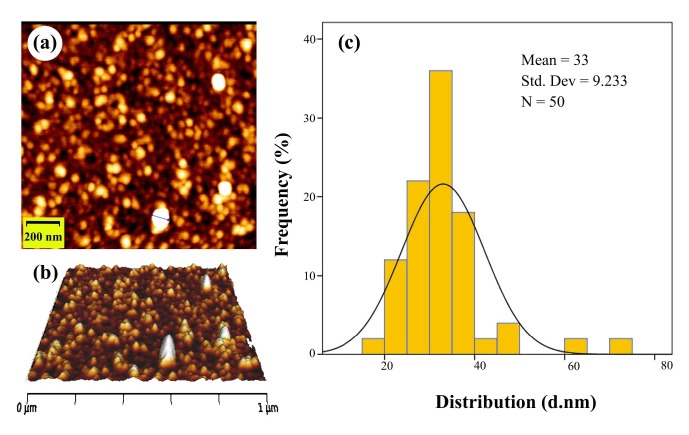
AFM image of TeNPs produced by *P. chrysogenum *PTCC 5031: (a) Two-dimentional image; (b) three-dimentional image; (c) size distribution

**Figure 4:**
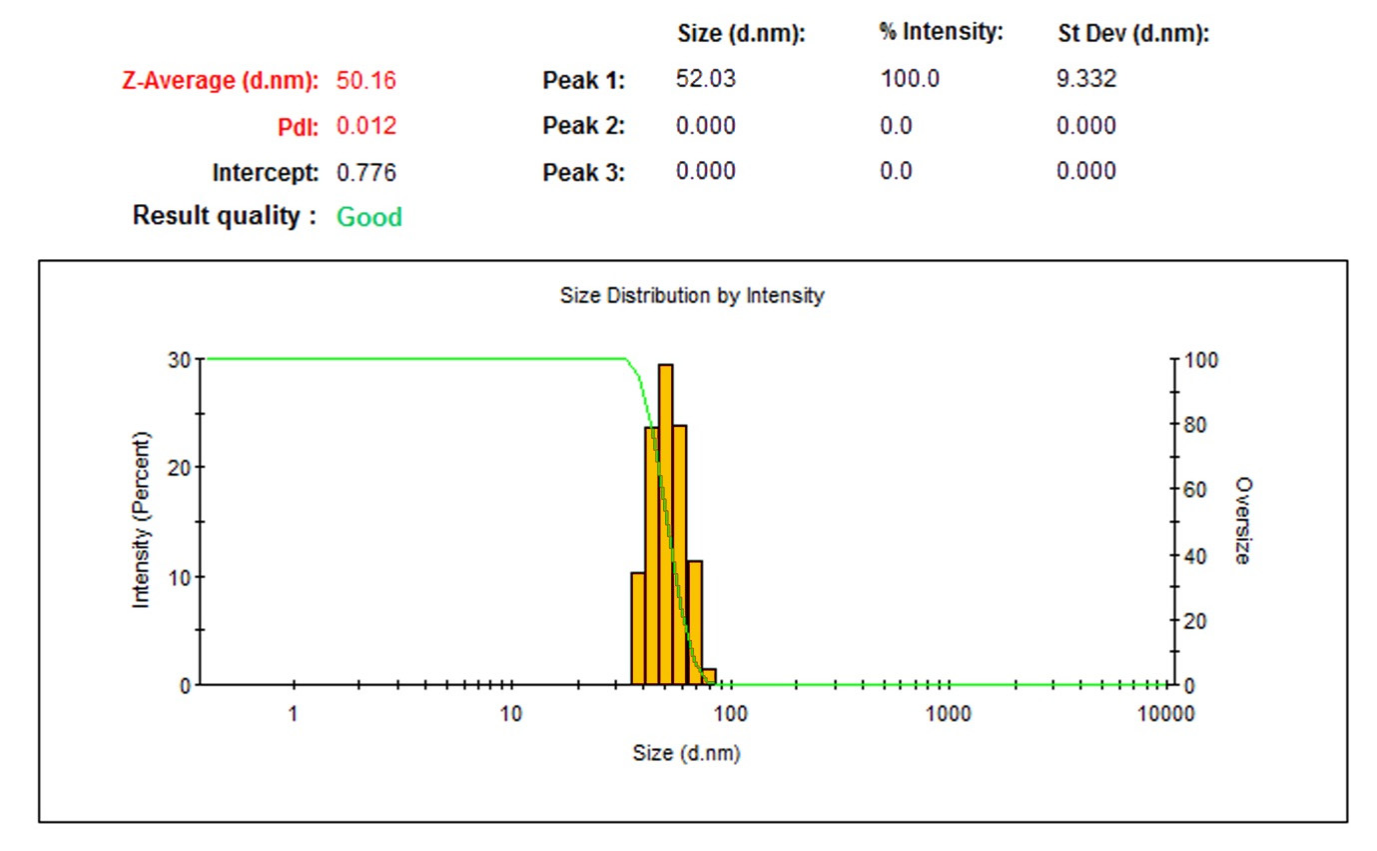
DLS of TeNPs produced by *P. chrysogenum *PTCC 5031

**Figure 5:**
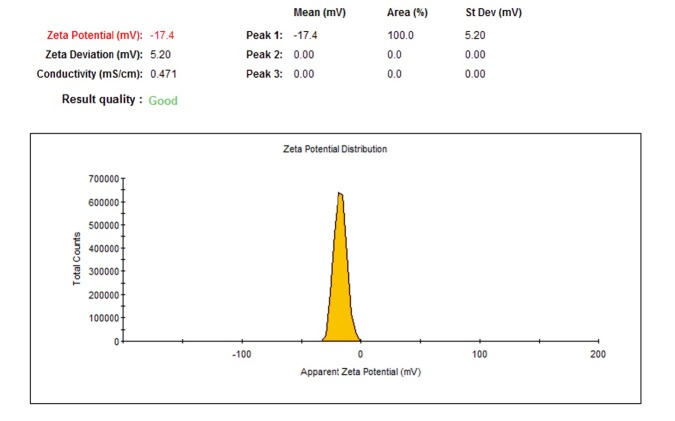
Zeta potential of TeNPs produced by *P. chrysogenum *PTCC 5031

**Figure 6:**
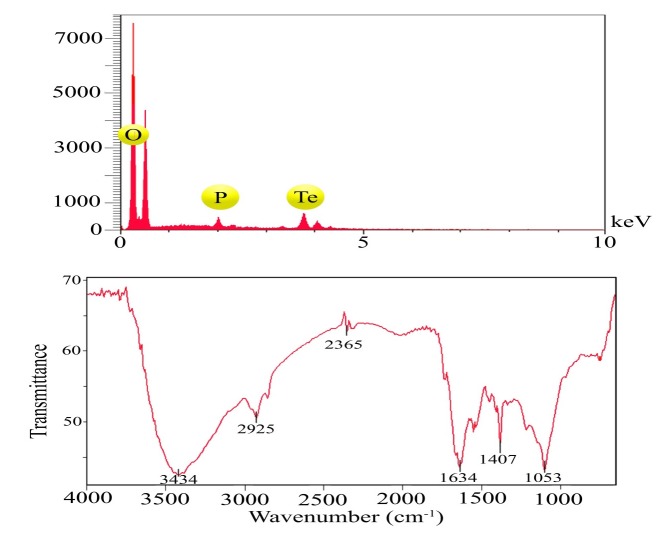
EDX (a) and FT-IR spectrum (b) of TeNPs produced by *P. chrysogenum *PTCC 5031

The macroscopic observations revealed gray black color in the reaction solution after five days of incubation following the addition of *P. chrysogenum* PTCC 5031 supernatant to colorless K_2_TeO_3_, 3H_2_O solution suggesting the formation of TeNPs, while the control sample containing Te^4+^ ions without extracellular secreted biomolecules and enzymes by fungus represented no change in color when incubated under the same conditions (Figure 1). More interestingly, a Tyndall effect was observed by using a laser beam that illuminated its path through the colloidal sample containing TeNPs, while no Tyndall effect was observed in the control sample solution contained tellurium ions (Figure 1c). Thereby, the Tyndall effect clearly confirmed the colloidal nature of biologically synthesized TeNPs. Additionally, the SEM micrograph exhibited that the TeNPs were spherical shaped and well distributed in the solution with average diameter of 33.80 nm (Figure 2). Interestingly, AFM micrograph showed quite agreeable results to SEM observations and confirmed that the nanoparticles were spherical in shape with good monodispersity with average diameter of 33 nm (Figure 3). Furthermore, Figure 4 represented the DLS analysis of TeNPs in the range of 35-85 nm with an average hydrodynamic diameter of 50.16 nm and polydispersity index (PdI) of 0.012 authenticating well-defined dimensions and good monodispersity of TeNPs. Besides, the zeta potential of the TeNPs was found at -17.4 mV (Figure 5). Moreover, the EDX results showed that TeNPs display an absorption peak at 3.8 keV indicating the presence of the elemental tellurium (Figure 6a). The EDX also depicted the presence of oxygen and phosphorous in the composition of biogenic NPs. Remarkably, the FT-IR spectrum of fungus-mediated synthesized TeNPs exhibited that nanoparticles manifests absorption peaks located at about 1053, 1407, 1634, 2365, 2925, and 3434 cm^-1^ in the region 450-4000 cm^-1^. The peaks at 3434 and 1634 cm^-1^ were assigned to O-H and C–C stretching, respectively. The band at 2925 cm^-1^ was associated with the C–H stretch of the methylene groups of the protein. Furthermore, the band at 1407 cm^-1^ corresponds to the N–H bending of primary amides due to carbonyl stretch in proteins. In addition, the peak at 1053 cm^-1^ could correspond to the C–N stretching vibration of the amine (Figure 6b). 

## Discussion

Nanomaterials are prepared via two approaches including top down and bottom up. In top down approach, nanomaterials are prepared from bulk materials using mechanical equipments, while in the bottom up approaches nanomaterials are prepared from the molecular level (22, 32, 33). Microbial-mediated synthesis of NPs is carried out through a bottom up approach. Practically, in this green process, NPs are synthesized intracellularly or extracellularly. The extracellular pathway has received more attention because of significant superiority over intracellular pathway owing to the fact that drawing out the NPs from cells may increase the risk of impurities via chemical reactions, while in extracellular process, NPs are prepared by utilization of extracellular enzymes and proteins in the reaction medium (30, 34, 35). Notably, it is a significant challenge to synthesize the NPs with desired shape and size. Interestingly, it is now well known that type of microorganism, growth medium, and synthesis conditions have a major influence on the size, shape, and monodispersity (36). 

In the current study, while the color of control remained unchanged during the entire incubation period, the successive biofabrication of TeNPs was apparent from the gradual alteration in the color of incubated solution from pale yellow to gray black indicated oxyanion reduction to its respective elemental form. This finding is in accordance with the results of other researchers (37, 38). Interestingly, different metallic nanoparticles display specific color change while converting to their nanostructures from their solution phase. For instance, gold, silver, and selenium exhibit purple (10, 39), brown (40, 41) and red-orange (42, 43) colors, respectively in their colloidal phase. Surface plasmon resonance (SPR) is the basis of this observation. Based on this phenomenon (SPR), the free electrons of colloidal metal nanoparticles begin to oscillate with specific energy. Hence, any wavelength of light are absorbed at that energy resulting in color change compared to the original metal color and/or metallic salt solution (13, 40). Additionally, one of the characteristic properties of colloidal systems is Tyndall effect that reveals the colloidal nature of the dispersion (44). In the current study, the colloidal nature of the biofabricated TeNPs in liquid phase was confirmed by Tyndall effect in which a laser beam passing through a colloidal solution leaves a discernible track due to light scattering. Other studies confirmed this simple and rapid test to verify the colloidal nature of the dispersed samples (45, 46). 

The SEM and AFM micrographs represented that biogenic TeNPs were spherical shaped along with well distribution (Figures 2 and 3). The PdI of 0.012 as an output of DLS analysis demonstrated good TeNPs monodispersity and confirmed microscopic observations. In details, PdI is a distribution index ranging from 0.01 to 1. The lower values refer to monodispersed samples, while upper values over 0.7 refer to polydispersed samples. Additionally, DLS depicted an average hydrodynamic diameter of 50.16 nm with zeta potential of -17.4 mV supplying the repulsive force as an electrostatic stabilization. The EDX analysis was conducted to determine elemental composition of the biogenic TeNPs that displayed a signal at 3.8 keV characteristics of elemental tellurium. It is of high importance to say that pH and electrolyte concentration significantly affect on zeta potential. Moreover, a colloidal system is electrostatically stable while the zeta potential is higher than + 30 mV or lower than -30 mV (13), thereby, the zeta potential of -17.4 mV in the current study suggested that another factor have a role to stabilize this colloidal system along with electrostatic stabilization. To justify this, it is suggested that steric stabilization resulting from complex of secreted enzymes and biomolecules from *P. chrysogenum *with TeNPs provide this stabilization. In chemical approach for synthesis of nanoparticles, chemical stabilizers are added to provide this steric stabilization to avoid their aggregation (47). No necessity for external stabilizers is a superiority of biological approach for preparation of nanoparticles (13). Interestingly, FT-IR spectrum showed the presence of biomolecules and functional groups on the surface of TeNPs that have a role not only as reducing agents to convert Te^4+ ^to Te^0^, but also as capping agents to avoid their aggregation. Previous studies reported that the enzyme nitrate reductase is in charge of bioreducing metal ions to form nanoparticles in the process of extracellular biofabrication of nanoparticles (5, 48). Up to now, several researchers have biosynthesized TeNPs with different shapes and sizes with both intracellular and/or extracellular pathways by employing bacteria. For instance, *Bacillus selenitireducens* fabricated rod shaped TeNPs on its surface with the size of around 10 nm (49). Besides, *Rhodobacter capsulatus* intracellularly synthesized rod shaped TeNPs with the size of around 100 nm (50). Likewise, *Pseudomonas pseudoalcaligenes* KF707 fabricated spherical TeNPs with the size of around 30 nm in an intracellular pathway. Interestingly, a study reported not only intracellular biosynthesis of rod shape TeNPs, but also evaluated its antibacterial activity against clinical isolates including *Staphylococcus aureus*, *Pseudomonas aeruginosa*, *Salmonella typhi*, and *Klebsiella pneumonia*, with a minimum bactericidal concentration (MBC) ranging from 125 to 500 µg/mL (51). Additional downstream processing needs to release the trapped TeNPs from inside the cells in intracellular pathway. Among microorganisms, fungi have been well considered for extracellular production of NPs owing to its advantages such as secretion of high amount of extracellular enzymes and proteins, and economic livability. Besides, compared to bacteria, facility of employing fungal biomass due to its filamentous structure is a superiority of exploiting fungi for biosynthesis of NPs (30). Although the extracellular production of NPs from several fungi have been reported up to now, some of them are pathogenic and the use of their biogenic NPs for pharmaceutical applications may have some risks. For instance, biosynthesis of NPs from *Aspergillus niger* has been reported (52, 53). On the other hand, based on evidence, *A. niger *strains produce mycotoxins (54). Consequently, it is of urgent need to explore medically safer fungi to biofabricate NPs for biomedical usages. Remarkably, *Penicillium* is a saprophyte family of fungi with a universal distribution that are used to produce cheeses as well as fermented sausages. Moreover, penicillin as an antibiotic was a product of this genus that had a significant role on the quality of life (55). Thereby, this family of fungi is of interest to find new candidate to produce NPs. For instance, Saxena *et al* 2017 reported extracellular synthesis of silver NPs induced by* P. chrysogenum* strain FGCC/BLS1 with an average particle size of 96.8 nm with strong antibacterial activity (56). Likewise, Sheet *et al* 2017 reported extracellular biosynthesis of silver NPs by using *P. chrysogenum* with significant antibacterial activity and cytotoxic effect against cancer cells (57). The present paper for the first time evaluated the ability of extracellular biosynthesis of TeNPs by using *P. chrysogenum*.

## Conclusion

In summary, the present study clearly demonstrated the extracellular biosynthesis of biogenic TeNPs via an eco-friendly and simple approach by exploiting enzymes and biomolecules secreted from *P. chrysogenum* PTCC 5031 at room temperature for the first time. Subsequently, biogenic TeNPs were characterized by SEM, AFM, DLS, EDX, and FT-IR. Interestingly, *Penicillium *species are widespread present in soils, foods, and indoor air with a worldwide distribution. Although biological preparation of NPs are in laboratory scale now, this available natural source gives an opportunity to cost-effective synthesis of not only TeNPs in this study, but also other nanoparticle-based theranostic agents involving quantum dots, silver, gold, and iron oxide NPs for biomedical and pharmaceutical applications. Further researches are required to evaluate the pharmaceutical potentials of biogenic TeNPs. It is expected that biogenic theranostic NPs may emerge as potential nanomedicine alone or in combination with FDA-approved drugs in the future. 
